# Social Activity and Cognitive Functioning Over Time: A Coordinated Analysis of Four Longitudinal Studies

**DOI:** 10.1155/2012/287438

**Published:** 2012-09-09

**Authors:** Cassandra L. Brown, Laura E. Gibbons, Robert F. Kennison, Annie Robitaille, Magnus Lindwall, Meghan B. Mitchell, Steven D. Shirk, Alireza Atri, Cynthia R. Cimino, Andreana Benitez, Stuart W. S. MacDonald, Elizabeth M. Zelinski, Sherry L. Willis, K. Warner Schaie, Boo Johansson, Roger A. Dixon, Dan M. Mungas, Scott M. Hofer, Andrea M. Piccinin

**Affiliations:** ^1^Department of Psychology, University of Victoria, P.O. Box 3050 STN CSC, Victoria, BC, Canada V8W 3P5; ^2^Harborview Medical Center and General Internal Medicine, University of Washington, Box 359780, 325 Ninth Avenue, Seattle, WA 98104, USA; ^3^Department of Psychology, California State University, Los Angeles, 5151 State University Drive, Los Angeles, CA 90032, USA; ^4^Department of Food and Nutrition, and Sport Science and Department of Psychology, University of Gothenburg, P.O. Box 100, SE 405 30 Gothenburg, Sweden; ^5^Department of Psychology, University of Gothenburg, P.O. Box 500, SE 405 30 Gothenburg, Sweden; ^6^Bedford Veterans Affairs Medical Center, Massachusetts General Hospital, Harvard Medical School, 200 Springs Rd, Bedford, MA 01730, USA; ^7^Department of Psychology and Neurology, University of South Florida, 4202 E. Fowler Avenue, Tampa, FL 33620, USA; ^8^Center for Biomedical Imaging, Medical University of South Carolina, 68 President St, MSC 120, Charleston, SC 29425, USA; ^9^Andrus Gerontology Center and Leonard Davis School of Gerontology, University of Southern California, Los Angeles, CA 90089-0191, USA; ^10^Department of Psychiatry and Behavioral Sciences, University of Washington, 180 Nickerson, Suite 206, Seattle, WA 98109, USA; ^11^Department of Psychology, University of Alberta, P-217 Biological Sciences Building, Edmonton, AB, Canada T6G 2E9; ^12^Davis Lawrence J. Ellison Ambulatory Care Center, University of California, 4860 Y Street, Ste 0100, Sacramento, CA 95817, USA

## Abstract

Social activity is typically viewed as part of an engaged lifestyle that may help mitigate the deleterious effects of advanced age on cognitive function. As such, social activity has been examined in relation to cognitive abilities later in life. However, longitudinal evidence for this hypothesis thus far remains inconclusive. The current study sought to clarify the relationship between social activity and cognitive function over time using a coordinated data analysis approach across four longitudinal studies. A series of multilevel growth models with social activity included as a covariate is presented. Four domains of cognitive function were assessed: reasoning, memory, fluency, and semantic knowledge. Results suggest that baseline social activity is related to some, but not all, cognitive functions. Baseline social activity levels failed to predict rate of decline in most cognitive abilities. Changes in social activity were not consistently associated with cognitive functioning. Our findings do not provide consistent evidence that changes in social activity correspond to immediate benefits in cognitive functioning, except perhaps for verbal fluency.

## 1. Introduction

Cognitive decline in older adulthood remains an area of great concern as the population ages. Some changes in cognitive function, such as decreased processing speed, are considered normative aspects of the aging process [1]. However, the impact of even mild cognitive impairment on functional capacity highlights the importance of maintaining cognitive function for as long as possible [2]. Substantial evidence suggests that lifestyle factors and cognitive function in older adulthood are related [3]. Sometimes summarized by the adage “use it or lose it,” current evidence suggests that leading an active lifestyle “using it” may buffer the effects of age-related cognitive decline “losing it” [3–5]. The mechanisms by which an active and engaged lifestyle may be related to better or preserved cognitive function in older adulthood remain to be fully elucidated. However, the cognitive reserve hypothesis predicts that some individuals are better able to withstand the physiological insults to the brain without measurable cognitive deficits because they had greater capacity to begin with [6]. Individuals may be able to actively increase their “reserve” through engaging in cognitively stimulating activities [3].

 Social activities are considered part of what constitutes an active and engaged lifestyle, alongside cognitive and physical activities [3, 4, 7–9]. However, the evidence for a relationship between social activity participation and cognitive function is mixed. Some studies have found a relationship between social activities and cognitive function [10], while others have failed to do so [11]. In an intervention study, older adults living in residential care with normal cognitive function, after participation in daily short duration social and physical activity sessions, performed better cognitively than they had during their baseline assessment. However, the study design confounded physical and social activity [12]. Conversely, Aartsen et al. [11] found no relationship between social activities and cognitive function six years later using a cross-lagged regression approach that attempted to elucidate the strongest causal pathway. Similarly, Green et al. [13] did not find support for the hypothesis that social contact is protective against later cognitive decline.

The inconclusiveness of results has been acknowledged previously and suggested to relate to differences in statistical techniques [4, 8]. However, among studies that have used similar analytical methods, such as logistic regression, results are still mixed (e.g., [14–17]). Research examining whether social engagement can predict cognitive function and change in cognitive function over time, using growth modeling techniques, has also lacked a consistent finding. James and colleagues [10] found that social activity at baseline was associated with a higher baseline level of global cognitive function and lower rate of decline. Ertel et al. [18] found that baseline social integration was associated with a slower rate of memory decline, but not baseline memory performance. In a study of Danish twins, McGue and Christensen [19] found that social activity at baseline was related to level of cognitive function but not to change in cognitive function over time. They also found that, within monozygotic same-sex twin pairs, there was no evidence that the more socially active twin was less likely to experience cognitive decline than the less active cotwin. Overall, these studies demonstrate that many questions remain about the relationship between social activity and cognitive function [4, 7].

It is not clear whether it is a lifetime of social engagement (the effects of social activity accrued over time) that is protective, or if changes in social activity are related to cognitive function. Small and colleagues [20] examined the relationship between changes in social activity and changes in cognitive function and found stronger evidence for changes in cognitive function predicting changes in social activity than the reverse. Other studies have included only baseline social activity as predictor. Yet, social relations have been found to change qualitatively as people age [21]. Changes in social activity at any point in time may correlate with changes in cognitive functioning after controlling for its overall trend. 

 The current paper builds on previous work by exploring whether including social activity as a variable that changes over time can clarify the relationship between social activity and cognitive function in older adulthood. Using multilevel growth modeling with social activity as a time-varying covariate allows an examination of the relations between time-specific changes in cognition and social activity. This allows for a detailed test of whether the impact of social activity on cognitive function is accrued over time or whether changing social activity levels might relatively quickly impact cognitive function. Examining the temporal relationship between social engagement and cognitive function is needed to inform theories of possible mechanisms.

 The current analysis examines the relationship between social activity, change in social activity, and four domains of cognition including: reasoning, memory, fluency, and semantic knowledge in four different populations. The same models are tested with data from four different longitudinal studies: the Long Beach Longitudinal Study (LBLS), the Seattle Longitudinal Study (SLS), the Victoria Longitudinal Study (VLS), and the Origins of Variance in the Oldest-Old: Octogenarian Twins Study (OCTO-Twin). This addresses the possibility that differing analytical methods may produce differing results and provides the opportunity for immediate replication and direct comparison of results. The diversity of the samples, two American, one Canadian, and one Swedish, increases the generalizability of the results and decreases the possibility that findings might be due to the particular features of one country or community. 

## 2. Method

This research, initiated as a partnership between the Advanced Psychometric Methods Workshop series (Mungas et al., NIA conference grant) and the Integrative Analysis of Longitudinal Studies on Aging (IALSA) network [22], brought workshop participants together with researchers from four IALSA member studies. These studies were specifically selected based on their collection of cognitive, physical, and social activity data along with a range of cognitive functioning measures over multiple occasions held in common across the four studies. While the activity and cognitive functioning variables are not always identical, the subsets of variables in each study were chosen based on the rationale that they tapped similar domains at the construct level (e.g., Fluid Reasoning (Gf), Crystallized Knowledge (Gc), Short-term Memory (Gsm), and Long-term Storage and Retrieval (Glr; e.g., category fluency)) [23]. In some cases the measures are the same, but more often they differ, providing opportunities for both strict and conceptual replication. An exception to this is the OCTO-Twin dataset, for which a fluency measure was not available. 

### 2.1. Origins of Variance in the Oldest-Old (OCTO-Twin) Participants (Sweden)

 The OCTO-Twin study is based on the oldest cohort of the Swedish Twin Registry and includes 702 participants aged 80 years and older at the time of the first assessment. Beginning in 1991–1993, the longitudinal design included a maximum of five measurement occasions at 2-year intervals. All individuals with a dementia diagnosis at baseline were excluded from the analyses (*n* = 98). The total sample includes 604 individuals, of whom 524 had the social activity measure and at least one of the cognitive measures. Approximately 20% of the sample was lost to followup at each wave (10% per year), but most of this attrition was due to death. The ratios for gender, education, socioeconomic status, marital status, and housing of the OCTO-Twin sample correspond to population statistics for this age range of the Swedish population [24]. Demographic information for the sample appears in Table 1.

#### 2.1.1. OCTO-Twin Measures and Procedure

**OCTO-Twin Cognitive Measures**

*Reasoning* was assessed using Koh's Block Design Test [25]. In this task, participants are presented with red and white blocks and several patterns on cards and asked to construct the design on the card with the blocks. *Memory* was assessed using the Prose Recall test in which participants are asked for immediate free recall of a brief (100 words) story that has a humorous point [26]. Responses are coded for the amount of information recalled in a manner similar to the Wechsler Memory Scale [27]. *Semantic knowledge *was assessed using the Swedish version of the Information Task [28], which includes questions of general knowledge.

**OCTO-Twin Social Activity**

Participants were asked at each wave: “*How many people do you see?*” The possible response was: “none” (0); “1-2” (1); “3–5” (2); “6–10” (3), or “11 or more” (4).

### 2.2. Long Beach Longitudinal Study Participants (CA, USA)

The LBLS was initiated in 1978 when participants were recruited from the Family Health Plan Health Maintenance Organization (HMO), including mainly residents of Long Beach and Orange County. This first panel included 583 individuals aged 28–36 or 55–87. The ethnic composition of the older group (98% Caucasian) was similar to the 65+ population for the area based on the 1970 census. Panel 2, initiated in 1992, included 633 individuals contacted from the same HMO (64 were excluded due to frank dementia or serious sensory or neurological problems). 

In order to include the same measures as those in the Seattle Longitudinal Study, LBLS Panel 1 (*n* = 106) and Panel 2 (*n* = 631) data from 1994 to 2003 were used in the current analysis, excluding individuals younger than age 55 in 1994 (baseline *n* = 565). During this period, data were collected at 3-year intervals. Attrition was approximately 50% over each interval, or 17% per year. Dementia incidence is not known. Descriptive information for the sample is presented in Table 2. Additional information on the LBLS design, measures, and participants can be found elsewhere [29, 30].

#### 2.2.1. LBLS Measures and Procedure

**LBLS Cognitive Measures**

*Reasoning* was assessed using a composite score of the Schaie-Thurstone Adult Mental Abilities Test (STAMAT; Schaie, [31]) Letter and Number Series tests. In Letter Series, participants view a series of letters (e.g., a b c c b a d e f f) and are asked to discover the rule that governs the series by identifying the letter that should come next in the series. Participants were to complete as many of the 30 items as possible within six minutes. Word Series was a parallel test to Letter Series but the letters were replaced with months (e.g., January) and days of the week (e.g., Monday). *Memory* involved immediate written recall of a list of 20 concrete high-frequency nouns studied for 3.5 minutes. *Fluency* was assessed by a word fluency task where participants were instructed to write down as many words as possible in five minutes that begin with the letter “s.” Participants were instructed that they could not use proper nouns or create words by changing endings of other listed words (e.g., if the letter was “w” and you already said “want,” you should not also say “wants,” “wanting,” or “wanted”). *Semantic knowledge* consisted of the STAMAT Recognition Vocabulary test. Participants were given a word and asked to circle a synonym of that word from four possible alternatives. The test included 50 items that were to be completed in five minutes.

**LBLS Social Activity Measure**

A measure of social activity was derived from a modified version of the Life Complexity Scale that was originally developed for the Seattle Longitudinal Study [32]. Participants were asked to record the number of “hours per week on average” they spent doing various activities (e.g., “going to parties”). The LBLS version of the scale included 34 specific activities, 7 of which were considered social. In the current study, these activity measures were dichotomized in order to distinguish those who reported no activity (coded as 0) from those who reported one or more hours of activity per week (coded as 1). This was done because the range of scores varied greatly within and between measures, and because some scores were highly deviant (skewed) from expected values (e.g., reporting more than 100 hours of reading per week).

The social activity variable was created by selecting questions from the Life Complexity Scale that fit the social activity construct. That is, a composite score was formed by summing up the number of social activity items that were endorsed as having one or more hours of activity per week. The measure consisted of seven questions including: phone conversations, voluntary activities, going to parties, going to dances, playing cards, visiting others, and attending church. The range of possible scores was 0 to 7. A social activity change variable was computed by subtracting the social activity measure in 1994 from social activity in 1994, 1997, 2000, and 2003. This resulted in a difference score that references the baseline testing in 1994.

### 2.3. Seattle Longitudinal Study Participants (WA, USA)

The SLS is a very long-running longitudinal study initiated by Schaie, who first recruited members of a local Health Maintenance Organization in 1956 [33]. Current analyses used up to four waves of SLS data from 1984 to 2005, which include an expanded set of measures that also overlap with the LBLS. Only participants of 55 years and older at baseline were included in the analysis. Baseline was defined as each participant's first study visit, and time was measured in all analyses as years in study (coded as 0, 7, 14, and 21). Attrition during these 7-year intervals was approximately 50%, or 7% per year. Dementia prevalence and incidence are not known. See Table 3 for SLS participant characteristics over the four waves of data analyzed here.

#### 2.3.1. SLS Measures and Procedure

**SLS Cognitive Measures**

In order to model roughly equivalent cognitive outcomes across the four studies included in this coordinated effort, our analysis included measures of reasoning, fluency, memory, and semantic knowledge from a larger battery of tests. *Reasoning* was assessed with the Word Series test from the Schaie-Thurstone Adult Mental Abilities Test (STAMAT [31]), in which participants were asked to determine a rule that governs a series of words (months or days of the week) by identifying what word should come next in a given series. Participants were provided with a printed word series and instructed to choose the next word in the series in multiple-choice format. The test consists of 30 items and total score is based on number of correct responses completed in 6 minutes. As in LBLS, *Fluency *was assessed with the word fluency test from the Primary Mental Abilities test [34]. *Memory* was assessed with a task in which participants were asked to study a list of 20 printed words for 3.5 minutes and provide immediate written recall of the items. *Semantic knowledge *was assessed with the Educational Testing Service (ETS) test of Advanced Vocabulary, in which participants were asked to identify synonyms for printed words from 5 choices [35]. Total score was based on number of correctly identified synonyms out of 36 test items completed within 4 minutes.

**SLS Social Activity Measure**

We followed the methodology described in the LBLS method portion of this paper in order to generate a roughly equivalent index of social activity (see LBLS section for a detailed description). Following this methodology, we created a composite activity measure by summing dichotomized responses from a modified version of the Life Complexity Scale [32] creating a seven-item social activity composite (volunteering, playing cards, phone conversations, visiting others, attending church, dancing, and partying). *Social activity change* was computed by subtracting baseline activity from each follow-up activity measure.

### 2.4. Victoria Longitudinal Study Participants (Victoria BC, Canada)

The Victoria Longitudinal Study began in 1986-1987 with a sample of 484 community residing volunteers and three-year retest intervals. Using a longitudinal sequential design, second and third independent samples began in 1992-1993 (*n* = 530) and 2001-2002 (*n* = 550) [36]. Each sample is tested at three-year intervals. To date, Sample 1 has been tested on seven occasions (over 18 years), Sample 2 on five (over 12 years), and Sample 3 on two occasions (over 6 years). Participants in all three samples were recruited between the ages of 55 and 85 years. 

Data from seven waves of Sample 1 and five waves of Sample 2 were included in the current investigation. Characteristics of the subsample analyzed here are provided in Table 4. Approximately 25% of the sample was lost to followup at each wave, or 8% per year. Dementia prevalence and incidence are not known. Further detail on the VLS design, measures, and participants can be found elsewhere [36].

#### 2.4.1. VLS Materials and Procedure

**VLS Cognitive Ability Measures**

*Fluency* was measured by performance on a Similarities task [35]. In this timed task, participants were presented with target words and asked to write as many words as possible with the same or nearly the same meaning within six minutes. *Memory *was indexed using a 30 item, noun list learning task comprised of five semantic categories. Participants studied the word list for two minutes followed by a five minute free recall task [37]. *Reasoning* was indexed by Letter Series [35] in which participants were presented with a series of letters and asked to identify the next letter in the sequence that was consistent with the sequence rule. Participants were given six minutes to complete the task*. Semantic knowledge* was assessed using a 54-item recognition vocabulary test. This task was adapted from the ETS Kit of Factor Referenced Tests [35].

**VLS Social Activity/Lifestyle Measure**

The social activity/lifestyle measure used in the presented investigation included a subset of items from the VLS Activity Lifestyle Questionnaire. Individual item distributions were reviewed and a small number of poorly distributed items were eliminated. Seven items were selected due to their social nature (Eat at restaurants, visit friend/relative, give dinner party, attend church, meetings of service organizations, meetings of clubs, and do volunteer work). For each item, participants indicated the frequency of engagement in that activity over the past two years on a scale from 0 to 9 (i.e., *never, less than once a year, about once a year, 2 or 3 times a year, about once a month, 2 or 3 times a month, about once a week, 2 or 3 times a week, daily*).

### 2.5. General Analytic Approach

The current analysis was conducted as part of a larger effort to examine the effects of lifestyle activities on cognitive function using the same analytic approach across studies from the Integrative Analysis of Longitudinal Studies on Aging (IALSA) network [22]. Thus, final models were selected in part to maintain consistency across lifestyle activities.

In order to improve ease of interpretation of our results, age, education, and social activity measures were mean centered for each study. The means for baseline age, education, and social activity were subtracted from their baseline values for each individual. This centered the covariates so that the intercept and linear slope terms would be interpreted as the expected value for an individual at the mean age and with the mean level of education for the study. The reference category for sex was male.

 In order to examine the effects of social activity on cognition, a series of multilevel models was fit with social activity as a baseline and a time varying covariate [38], with time specified as time since baseline, using multilevel mixed-effects regression in StataCorp [39],the restricted maximum likelihood estimator (REML), and an unstructured covariance matrix. In the OCTO-Twin study, participants were nested within their twin pair. In the VLS, we controlled for enrolment cohort. Model assumptions were verified by examining the residuals. Separate models were fit for each of the four cognitive measures (reasoning, fluency, memory, and semantic knowledge) resulting in four reported models for each study. While the “familywise” alpha rate within each individual study may be somewhat liberal, given our use of *P* < .05 as significance criterion, our focus on repetition of findings across studies imposes a strict limit to any reliance on chance findings. Formal meta-analytic methods, which would require identical measures and a larger number of studies, were not used. Instead we relied on comparison of the conclusions derived from each study.

 An initial 19-term model included the following terms: (1) baseline age, (2) sex, (3) education, (4) baseline social activity, (5) baseline social activity × age, (6) baseline social activity × sex, (7) baseline social activity × education, (8) individually defined time since baseline, (9) time × baseline age, (10) time × sex, (11) time × education, (12) time × baseline social activity, (13) time × baseline social activity × baseline age, (14) time × baseline social activity × sex, (15) time × baseline social activity × education, (16) change in social activity from baseline (activity change), (17) social activity change × baseline age, (18) social activity change × sex, and (19) social activity change × education. However, several terms were not significant for most of the studies and outcomes and so were trimmed to facilitate model interpretation. This process eliminated 7 of the 19 terms, first the 3-way interactions were eliminated, then the interactions with change in social activity. Last, the baseline social activity by sex interaction was dropped. This resulted in a 12-term final model, presented in Table 5 for separate cognitive constructs of reasoning, memory, semantic knowledge, and fluency.

## 3. Results

Significant between person age differences were seen at the first occasion of measurement for all memory, reasoning, and fluency tests, with older adults performing less well than their younger counterparts (all *P* < .01). Semantic knowledge results were less consistent, with SLS showing no age differences, *b* = 0.02, *P* = .35, LBLS and OCTO-Twin suggesting the older individuals score worse, *b* = − 0.28, *P* < .01, and *b* = − 0.55, *P* < .01, respectively, and VLS finding that older individuals performed slightly better, *b* = 0.08, *P* = .02. 

At baseline, individuals with more years of education had significantly higher cognitive performance on all tasks, across all studies (all *P* < .01). Women at the mean baseline age had higher memory scores than did same aged men across all studies (all *P* < .01). LBLS and SLS women scored higher than men on all measures (reasoning: *P* = .02 and *P* < .01; fluency: both *P* < .01), except for LBLS semantic knowledge (*P* = .13, SLS *P* = .02). OCTO-Twin women had significantly lower Information scores, considered semantic knowledge, at baseline than OCTO-Twin men (*b* = − 4.20, *P* < .01).

Significant within person declines were seen over time in study in all cognitive abilities and all studies (all *P*s < .01). A significant time∗age interaction indicated that within each sample, those who were older at baseline declined significantly faster than the younger participants on all VLS, SLS, and LBLS measures except LBLS immediate memory. Except for the Information test, evidence for differential decline in older individuals was not seen in OCTO-Twin, which has a much narrower age range.

Women showed less decline than men in the SLS data only, and only for the fluency and semantic knowledge measures. Evidence for differential decline related to education was seen only for the LBLS reasoning measure.

Higher social activity levels at baseline, for individuals of average age and education, were associated with higher scores on reasoning within the VLS and OCTO-Twin studies, *b* = 0.05, *P* = .03, and *b* = 1.01, *P* < .01, respectively. The most consistent finding was that individuals with higher social activity levels at baseline also had higher memory scores. This was true for the SLS, VLS, and OCTO-Twin studies, *b* = 0.14, *P* = .01, *b* = 0.06, *P* < .01, *b* = 0.34, *P* < .01, respectively. Baseline social activity was also positively related to fluency in the LBLS (though the same measure was not significant in SLS sample). Social activity at baseline and semantic knowledge was positively related in the OCTO-Twin sample (operationalized as information; *b* = 0.87, *P* = .03), but negatively related in the SLS sample (operationalized as vocabulary; *b* = − 0.22, *P* = .02). The OCTO-Twin sample was the only one where all cognitive measures considered had a positive association with baseline social activity levels. 

The Baseline Age∗Social Activity interaction terms were significant and positive for LBLS reasoning (*b* = 0.08, *P* < .01) and semantic knowledge (*b* = 0.08, *P* < .01), indicating a stronger effect of social activity for older participants than for younger participants. However, the interaction of age and social activity was not significant for any other studies or cognitive measures (*Ps* > .05). The interaction between education and social activity at baseline was not significantly associated with any cognitive measure within any of the samples. 

In terms of within person age changes, social activity at baseline was significantly related to the slope of semantic knowledge for SLS participants such that higher social activity at baseline was associated with less semantic knowledge decline (*P* = .01). There were no other significant relationships between social activity at baseline and rate of change for any cognitive measure within any of the samples (all *P* > .05).

In examining associations with change in social activity as a time-varying covariate in each of the models, several significant relationships were found. A reported increase in social activity was positively associated with performance on the fluency measures in all studies except SLS (LBLS *P* < .01, VLS *P* < .01, SLS *P* = .21), such that individuals who increased their level of social activity from their own baseline level exhibited higher occasion-specific fluency performance relative to their expected linear trajectory over time. In VLS and SLS, a significant relationship was also found between change in social activity and the reasoning (VLS *P* = .02, SLS *P* = .04) and memory (VLS *P* < .01, SLS *P* = .03) measure, indicating that participants who increased their social activity level also scored higher relative to their expected trajectory on the reasoning or memory measure. Changes in social activity were related to all cognitive measures in the OCTO-Twin study (all *P* < .05), such that OCTO-Twin participants who increased their social activity level from baseline had higher occasion-specific cognitive scores relative to their own linear trajectory in all domains tested. 

## 4. Discussion

Comparing results of the same statistical model across four longitudinal studies of aging, we observed relatively few consistent associations between social activity and cognitive functioning. Looking at within person change in social activity and the four cognitive measures, the most consistent finding was an association with fluency in two of the three studies measuring it, after adjusting for linear effects of time and other covariates. Change in social activity was associated with memory performance in only half of the four studies (SLS and VLS). In only one study, OCTO-Twin, change in social activity was significantly related to performance in all three of the cognitive domains considered (fluency measure not available). The OCTO-Twin sample was the oldest and had the narrowest range of ages. Social activity in the OCTO-Twin study was defined by the number of people with whom participants had contact, whereas the other studies included a range of activities with a social component. Given that within person decreases in performance were seen across all cognitive measures in all studies, the positive relationships generally represent attenuated decline, rather than improvement in cognitive performance. 

The use of within person change in social activity as a time-varying predictor of cognitive function is somewhat unique. However, in another study examining the temporal relations of social activity change and cognitive function, Small and colleagues [20] found significant coupling of social activity change and three cognitive domains: semantic decision speed (similar to our fluency measure), episodic memory, and semantic memory. They, however, found greater support for models where cognitive measures predicted changes in social activity levels, than the reverse. 

There was little evidence that initial levels of social activity were related to within person rate of decline in cognitive function. Although higher initial levels of social activity were associated with less decline on the SLS semantic knowledge measure, this was not evident in any other samples. SLS has the widest interval between measurements and so correspondingly greater attrition between waves (though a similar yearly rate), but this does not suggest an obvious reason for the difference. The findings from the other studies are consistent with McGue and Christensen [19], who found that social activity at the first assessment was related to initial level of cognitive function but not change in cognitive function. However, other groups have found that social activity levels are associated with both baseline cognitive function and a reduced rate of decline over time [10]. Contrary to our findings, but similarly examining specific cognitive domains, Ertel et al. [18] found that social integration was related to a slower rate of memory decline. We did not include a composite measure of general cognitive function, but our results, and the results of others who have examined specific facets of cognition, suggest that it is important to examine cognitive domains separately. 

Several between person differences were found. Specifically, individuals with higher initial levels of social activity performed better on the memory measures in three of the four samples. Interestingly, the sample that did not show the effect was the LBLS, which used the same measure as the SLS study. This suggests the discrepancy is not a function of different memory measures being used, or how social activity was characterized in the study. It is perhaps related to differences in samples, although both were similar in average years of education and were American, although from different regions. It is possible that how well a measure captures social activity varies by community. This, and the accessibility of the activities, may influence the association with cognitive function. However, such differences would not likely be specific to memory performance. The mean age of LBLS participants was about six years older and the first wave of LBLS assessment included in the present study was conducted ten years later than the first assessment of the SLS. The age differences are not an obvious explanation, because the mean age of LBLS participants is only slightly older than SLS and VLS participants and is nearly ten years younger than OCTO-Twin participants. The LBLS is the only study for which the majority of participants are not female, though this difference is slim (49% female in the LBLS versus 52% in SLS at baseline). It is possible another factor, such as attrition, is playing a role. The LBLS does have the highest yearly rate of attrition (17% versus 10%, 7%, and 8%), although the loss at each measurement wave is similar to that of SLS. It is unclear how this would affect the association between baseline social activity and memory performance; however, particularly considering that the mean social activity and memory measure scores for the LBLS and SLS were similar. It would be interesting in future work to consider general health and its age gradient in each of the studies. The lack of obvious reasons for discrepant findings, however, supports the importance of considering the reproducibility of results in coordinated, rather than pooled, analyses.

Social integration has been posited to influence general health through multiple pathways that may overlap with those influencing cognition [40]. Unfortunately, our findings do not strongly suggest that increased engagement in social activities confers immediate benefit in terms of cognitive function. Nor do they suggest that social participation reduces risk of cognitive decline in any domain apart from fluency. The cognitive reserve hypothesis suggests that, over time, “reserve” can be built up through stimulating activities and that individuals with high reserve can withstand more physiological deterioration before cognitive decline is observable [6]. That social participation does not reduce the risk of cognitive decline in a broad range of measures suggests that it is not conferring “reserve.”

One possible mechanism through which social engagement and cognitive function are related may be the cognitively stimulating nature of social activities [20]. The cognitive training literature has found that training in specific tasks (e.g., memory tasks) does not necessarily transfer to other cognitive domains [3]. This may be similarly true of social activity participation, whereby a relationship is only seen in cognitive domains that are being challenged by the social activity. Social interactions do typically involve verbal communication, and thus fluency, likely specifically verbal fluency, may be the cognitive domain most similar to our participants' social activities. 

 Discrepancies in which activities are considered social may contribute to the lack of consistent association between social activity and cognitive function. For example, some activities (e.g., card games) may generally be more cognitively demanding than others (e.g., visits from family members), or primarily tax different cognitive skills, but this is likely to differ across individuals and situations. Although plausible, further research would need to confirm the validity of such hypothesis. In the context of physical health, others [40] have suggested that different social factors (e.g., social influence, social engagement, and social support) may act primarily through different behavioral, psychological, and physiological pathways. Similarly, cognitive function may be differentially influenced depending on the particular combination of social factors and pathways. In this way, how social activity is conceptualized and measured may be related to the mechanistic pathway and thus differentially related to various domains of cognitive function. These effects may have contributed to the discrepancies between OCTO-Twin and the other three studies in the current analysis, as OCTO-Twin focused on frequency of contact with people as the social activity measure, whereas the other three encompassed a variety of activities. However, the lack of consistency between the two studies employing the same measures suggests that conceptualization of social activity does not fully explain the findings. A clue to the source of inconsistency across LBLS and SLS may be the very small average change in the LBLS social activities measure, though its variance is comparable to those of SLS and OCTO-Twin. The association between cognition and social activity in LBLS in particular also seems to vary by age more than in the other studies.

A considerable strength of our analysis is the replication of the models across four longitudinal studies from geographically separate regions. This limits the possibility of spurious findings taking on undue importance and provides an opportunity to examine consistencies and inconsistencies between sample groups.

In terms of potential methodological limitations, Hertzog et al. ([41] for slope covariances; [42] for variance components) suggest that the power to detect correlated change is extremely low. However, analysis of longitudinal studies on aging, including those used in the current paper, has consistently reported statistically significant variances and covariances in rates of change in cognitive outcomes ([43–54], for summary see [55]). In addition, the tests used by Hertzog et al. are based on significance tests for variance components whereas change was evaluated in our paper by examining fixed effects which will generally have greater power [56]. Lending support to the argument that analyses in the current paper were adequately powered are the findings of significant associations between the identical set of cognitive outcomes in these same studies and physical and cognitive activities [57, 58].

Another limitation to our analysis is that while the study samples were restricted to initially healthy older adults, efforts were not made to exclude individuals who developed dementia over the course of the study periods (dementia diagnosis was available only in the OCTO-Twin study). We cannot assume that any protective effects of social activity on cognitive function would be equivalent across healthy and dementing older adults. Including individuals whose cognitive decline may have been driven by the dementia process may impact the associations between social activity and cognitive function over time in nondementing individuals, and this may also contribute to inconsistencies in the literature. 

Finally, it is difficult to rule out the possibility that individuals decrease their social activities because their declining cognitive abilities make it more difficult for them to maintain social ties. The difficulty of determining the directionality of the relationship has been well acknowledged in the literature and it is similarly difficult to determine causal pathways in the current analysis [4, 5, 10]. Some attempts have been made to determine the most likely direction of the relationship, but the evidence is limited [20]. The longitudinal nature of the studies considered here, and allowing social activity to vary across time, have narrowed the temporal distance between the social activity and cognitive performance, at least partially disentangling whether the relationship is primarily based on historical activity levels or whether the two tracks over time. 

Across four studies in three countries, baseline social activity levels failed to predict rate of decline in most cognitive abilities, and changes in social activity were not consistently associated with within person fluctuations in cognitive functioning. Our findings leave little support for the hypothesis that changes in social activity correspond to immediate benefits in cognitive functioning, except perhaps for fluency.

## Figures and Tables

**Table 1:** OCTO-twin participant characteristics.

	Year of testing				
Measure	Baseline (*n* = 524)	Year 2 (*n* = 424)	Year 4 (*n* = 326)	Year 6 (*n* = 245)	Year 8 (*n* = 175)
	M (SD)	M (SD)	M (SD)	M (SD)	M (SD)
Retention from previous testing (%)		82.9	76.7	76.2	72.8
Age	83.2 (2.9)	85.2 (2.8)	86.9 (2.5)	88.8 (2.5)	90.7 (2.4)
Education	7.2 (2.3)	7.3 (2.4)	7.3 (2.3)	7.2 (2.1)	7.2 (2.3)
Sex, female [*n* (%)]	365 (66)	299 (65)	233 (66)	193 (72)	146 (74)
Reasoning	11.6 (7.1)	11.6 (7.1)	11.5 (7.0)	10.2 (7.3)	10.2 (7.3)
Memory	9.6 (4.0)	9.5 (4.2)	9.5 (4.2)	9.1 (4.4)	9.1 (4.4)
Semantic knowledge	28.2 (11.1)	28.8 (11.1)	28.8 (11.1)	26.1 (11.4)	26.1 (11.4)
Social activity	3.0 (1.0)	3.0 (1.0)	3.1 (0.9)	2.8 (1.0)	2.8 (0.9)
Social activity change	—	−0.1 (1.0)	−0.0 (1.1)	−0.2 (1.1)	−0.4 (1.2)

M: mean, SD: standard deviation. The range for each measure with a defined upper limit is as follows: reasoning = 0–42, memory = 0–16, semantic knowledge = 0–44, and social activity = 0–4. Higher scores represent higher activity.

**Table 2:** LBLS participant characteristics.

	Year of testing			
Measure	Baseline (*n* = 565)	Year 3 (*n* = 300)	Year 6 (*n* = 143)	Year 9 (*n* = 102)
	M (SD)	M (SD)	M (SD)	M (SD)
Retention from previous testing (%)		53	48	71
Age	73.8 (9.1)	75.5 (8.7)	75.29 (8.01)	76.4 (7.2)
Education	13.7 (3.0)	13.9 (2.8)	14.2 (2.70)	14.1 (2.7)
Sex, female [*n* (%)]	278 (49)	148 (49)	74 (52)	50 (49)
Reasoning	22.1 (11.7)	23.7 (11.5)	25.2 (11.63)	25.0 (11.1)
Fluency	32.3 (11.7)	33.5 (11.1)	33.3 (13.29)	34.3 (11.7)
Memory	11.4 (4.0)	11.5 (4.2)	11.6 (4.52)	11.1 (4.7)
Semantic knowledge	38.4 (10.2)	39.3 (9.8)	40.8 (8.95)	39.4 (9.9)
Social activity	3.3 (1.5)	3.5 (1.6)	3.5 (1.4)	3.5 (1.5)
Social activity change	—	0.0 (1.4)	0.1 (1.5)	−0.0 (1.4)

M: mean, SD: standard deviation. The range for each measure with a defined upper limit is as follows: education = 0–20, reasoning = 0–30, memory = 0–20, semantic knowledge = 0–36, and social activity = 0–7.

**Table 3:** SLS participant characteristics.

	Year of testing			
Measure	Baseline (*n* = 1657)	Year 7 (*n* = 940)	Year 14 (*n* = 446)	Year 21 (*n* = 181)
	M (SD)	M (SD)	M (SD)	M (SD)
Retention from previous testing (%)		57	47	41
Age	67.1 (8.2)	73.0 (7.23)	78.0 (6.4)	81.9 (4.9)
Education	14.6 (2.9)	14.7 (2.8)	14.8 (2.7)	14.8 (2.8)
Sex, female [*n* (%)]	861 (52)	507 (54)	256 (57)	108 (60)
Reasoning	15.6 (5.8)	15.2 (5.6)	14.3 (5.5)	14.0 (5.25)
Fluency	38.5 (12.8)	37.5 (13.1)	36.7 (12.7)	38.8 (14.5)
Memory	12.5 (4.0)	12.0 (4.1)	11.5 (4.1)	11.6 (4.0)
Semantic knowledge	25.0 (6.7)	25.3 (6.6)	25.8 (6.2)	25.9 (5.9)
Social activity	3.5 (1.6)	3.5 (1.6)	3.5 (1.6)	3.3 (1.6)
Social activity change	—	−0.1 (1.5)	−0.2 (1.6)	−0.4 (1.7)

M: mean, SD: standard deviation. The range for each measure with a defined upper limit is as follows: education = 0–20, reasoning = 0–30, memory = 0–20, semantic knowledge = 0–36, and social activity = 0–7.

**Table 4:** VLS participant characteristics.

	Year of testing						
Measure	Baseline (*n* = 977)	Year 3 (*n* = 723)	Year 6 (*n* = 571)	Year 9 (*n* = 411)	Year 12 (*n* = 275)	Year 15 (*n* = 91)	Year 18 (*n* = 52)
	M (SD)	M (SD)	M (SD)	M (SD)	M (SD)	M (SD)	M (SD)
Retention from previous testing (%)^a^	—	74	79	72	67	79	57
Age	68.6 (6.7)	71.3 (6.6)	73.7 (6.4)	76.5 (5.9)	79.3 (5.2)	82.2 (4.6)	85.1 (3.6)
Years of education at baseline	14.9 (3.3)	15.4 (3.2)	15.6 (3.1)	15.9 (3.1)	15.8 (3.1)	15.2 (3.1)	14.8 (2.8)
Sex, female [*n* (%)]	614 (62.8)	450 (62.2)	346 (60.6)	251 (61.1)	170 (61.8)	60 (65.9)	35 (67.3)
Reasoning^b^	11.2 (4.7)	11.7 (4.2)	10.3 (4.7)	10.3 (4.6)	9.9 (4.6)	7.5 (4.7)	6.5 (4.2)
Fluency	17.7 (4.3)	17.9 (4.4)	17.7 (4.5)	17.2 (4.8)	16.3 (4.9)	14.7 (5.8)	13.8 (5.4)
Memory^c^	13.7 (5.9)	14.7 (6.0)	14.7 (6.1)	14.9 (6.4)	11.7 (5.4)	13.0 (6.3)	—
Semantic knowledge	43.7 (7.4)	44.7 (6.2)	44.3 (6.0)	44.2 (5.8)	43.5 (5.7)	42.7 (7.1)	42.6 (6.6)
Social activity	23.0 (6.9)	23.2 (7.2)	22.8 (7.3)	22.5 (7.2)	21.2 (7.1)	22.8 (7.7)	21.1 (7.2)
Social activity change	—	−0.1 (5.4)	−0.3 (6.0)	−0.9 (6.4)	−2.5 (6.9)	−3.5 (7.5)	−5.3 (8.1)

M: mean, SD: standard deviation. The range for each measure with a defined upper limit is as follows: reasoning = 0–20, Memory = 0−30, and Semantic knowledge 0−54.

^a^The 1986 cohort was followed for up to 18 years, the 1993 cohort for up to 12.

^b^The reasoning measure was not given until year 6 for the 1986 cohort.

^c^The memory measure was not given in year 18.

**Table 5:** Mixed model results of four longitudinal studies.

	OCTO-Twin			LBLS			SLS			VLS		
	*b*	SE	*P*	*b*	SE	*P*	*b*	SE	*P*	*b*	SE	*P*
	Reasoning											

Intercept	10.89	0.55	<.01	21.43	0.53	<.01	14.65	0.17	<.01	9.52	0.59	<.01
Age	−0.33	0.12	.01	−0.62	0.04	<.01	−0.35	0.01	<.01	−0.26	0.02	<.01
Female	0.68	0.67	.31	1.86	0.77	.02	1.57	0.24	<.01	−0.15	0.31	.62
Education	0.56	0.13	<.01	1.30	0.13	<.01	0.52	0.04	<.01	0.32	0.05	<.01
Social activity	1.01	0.28	<.01	0.08	0.25	.74	0.11	0.08	.14	0.05	0.02	.03
Age × social activity	−0.02	0.11	.83	0.08	0.03	<.01	0.01	0.01	.15	0.00	0.00	.40
Education × social activity	0.04	0.11	.71	−0.04	0.08	.67	−0.03	0.03	.26	−0.01	0.01	.69
Slope	−0.44	0.08	<.01	−0.50	0.08	<.01	−0.25	0.02	<.01	−0.25	0.03	<.01
Age	−0.02	0.02	.28	−0.03	0.01	<.01	−0.01	0.00	<.01	−0.01	0.00	<.01
Female	0.08	0.09	.42	0.08	0.11	.48	0.01	0.02	.51	0.01	0.03	.76
Education	0.01	0.02	.56	−0.05	0.02	<.01	−0.01	0.00	.11	−0.01	0.00	.08
Social activity	0.06	0.05	.24	0.04	0.04	.30	0.02	0.01	.02	0.00	0.00	.44
Social activity change	0.65	0.15	<.01	0.04	0.17	.83	0.13	0.06	.04	0.03	0.00	.02

	Fluency											

Intercept				31.01	0.61	<.01	36.11	0.44	<.01	11.5	0.56	<.01
Age				−0.30	0.05	<.01	−0.34	0.04	<.01	−0.10	0.03	<.01
Female				3.39	0.88	<.01	3.36	0.60	<.01	0.51	0.36	.16
Education				1.05	0.15	<.01	1.31	0.11	<.01	0.63	0.05	<.01
Social activity				0.99	0.29	<.01	0.24	0.19	.20	0.04	0.03	.12
Age × social activity				0.03	0.03	.40	0.00	0.02	.89	−0.01	0.01	.29
Education × social activity				−0.11	0.10	.24	0.04	0.07	.59	−0.01	0.01	.07
Slope				−0.24	0.11	.02	−0.43	0.04	<.01	−0.12	0.03	<.01
Age				−0.03	0.01	<.01	−0.02	0.00	<.01	−0.01	0.01	<.01
Female				−0.20	0.15	.18	0.11	0.05	.02	0.06	0.04	.16
Education				−0.01	0.03	.65	−0.01	0.01	.15	−0.01	0.01	.74
Social activity				0.07	0.05	.15	0.02	0.02	.26	0.01	0.01	.83
Social activity change				0.73	0.25	<.01	0.18	0.15	.21	0.06	0.02	<.01

	Memory											

Intercept	8.70	0.32	<.01	10.80	0.20	<.01	11.56	0.13	<.01	17.28	0.41	<.01
Age	−0.20	0.06	<.01	−0.19	0.02	<.01	−0.18	0.01	<.01	−0.19	0.02	<.01
Female	0.99	0.39	.01	1.50	0.28	<.01	1.59	0.17	<.01	1.45	0.27	<.01
Education	0.34	0.07	<.01	0.29	0.05	<.01	0.32	0.03	<.01	0.31	0.04	<.01
Social activity	0.34	0.07	<.01	0.00	0.09	.98	0.14	0.06	.01	0.06	0.02	<.01
Age × social activity	−0.09	0.06	.10	0.01	0.0	.61	0.00	0.01	.51	−0.01	0.01	.95
Education × social activity	0.01	0.06	.91	−0.02	0.03	.57	−0.02	0.02	.18	−0.01	0.01	.19
Slope	−0.26	0.07	<.01	−0.18	0.05	<.01	−0.17	0.01	<.01	−0.27	0.03	<.01
Age	0.00	0.02	.97	0.00	0.01	.46	−0.01	0.00	<.01	−0.01	0.01	<.01
Female	0.05	0.08	.55	−0.03	0.07	.64	0.02	0.02	.15	−0.02	0.03	.56
Education	−0.01	0.02	.73	0.01	0.01	.40	0.00	0.00	.58	−0.01	0.01	.15
Social activity	0.07	0.04	.07	0.03	0.02	.15	0.01	0.01	.37	0.01	0.01	.25
Social activity change	0.47	0.11	<.01	0.11	0.10	.30	0.12	0.06	.03	0.06	0.01	<.01

	Semantic knowledge											

Intercept	30.49	0.86	<.01	38.19	0.54	<.01	24.08	0.22	<.01	43.77	0.71	<.01
Age	−0.55	0.17	<.01	−0.28	0.04	<.01	0.02	0.02	.35	0.08	0.03	.02
Female	−4.20	1.05	<.01	1.19	0.78	.13	0.70	0.30	.02	−0.01	0.46	.99
Education	1.43	0.19	<.01	1.03	0.14	<.01	1.12	0.05	<.01	0.77	0.07	<.01
Social activity	0.87	0.40	.03	0.10	0.25	.70	−0.22	0.10	.02	0.02	0.03	.63
Age × social activity	−0.10	0.15	.47	0.08	0.03	<.01	−0.01	0.01	.61	0.01	0.01	.77
Education × social activity	0.03	0.17	.84	−0.06	0.09	.50	−0.05	0.03	.15	0.01	0.01	.61
Slope	−0.85	0.11	<.01	−0.46	0.08	<.01	−0.12	0.01	<.01	−0.16	0.03	<.01
Age	−0.05	0.02	.03	−0.04	0.01	<.01	−0.01	0.00	<.01	−0.01	0.01	<.01
Female	0.19	0.13	.15	0.12	0.12	.30	0.06	0.02	<.01	0.03	0.04	.42
Education	0.03	0.03	.29	−0.02	0.02	.32	0.00	0.00	.78	−0.01	0.01	.18
Social activity	0.11	0.07	.12	0.01	0.04	.95	0.01	0.01	.01	−0.01	0.01	.40
Social activity change	0.86	0.19	<.01	0.17	0.17	.33	0.08	0.06	.13	−0.01	0.01	.93

Values represent model coefficients and their standard error. Across all studies, time was measured in years since baseline visit and activity change was entered as a time-varying covariate. All other variables represent baseline measurements alone, in interaction with one another, or in interaction with time.
